# Non-Thermal Atmospheric Pressure Plasma Preferentially Induces Apoptosis in p53-Mutated Cancer Cells by Activating ROS Stress-Response Pathways

**DOI:** 10.1371/journal.pone.0091947

**Published:** 2014-04-23

**Authors:** Yonghao Ma, Chang Seung Ha, Seok Won Hwang, Hae June Lee, Gyoo Cheon Kim, Kyo-Won Lee, Kiwon Song

**Affiliations:** 1 Department of Biochemistry, College of Life Science & Biotechnology, Yonsei University, Seoul, Korea; 2 Department of Electrical Engineering, Pusan National University, Pusan, Korea; 3 Department of Oral Anatomy, School of Dentistry, Pusan National University, Yangsan, Korea; 4 Department of Obstetrics and Gynecology, Kangbuk Samsung Hospital, Sungkyunkwan University School of Medicine, Seoul, Korea; University of Florida, United States of America

## Abstract

Non-thermal atmospheric pressure plasma (NTAPP) is an ionized gas at room temperature and has potential as a new apoptosis-promoting cancer therapy that acts by generating reactive oxygen species (ROS). However, it is imperative to determine its selectivity and standardize the components and composition of NTAPP. Here, we designed an NTAPP-generating apparatus combined with a He gas feeding system and demonstrated its high selectivity toward p53-mutated cancer cells. We first determined the proper conditions for NTAPP exposure to selectively induce apoptosis in cancer cells. The apoptotic effect of NTAPP was greater for p53-mutated cancer cells; artificial p53 expression in p53-negative HT29 cells decreased the pro-apoptotic effect of NTAPP. We also examined extra- and intracellular ROS levels in NTAPP-treated cells to deduce the mechanism of NTAPP action. While NTAPP-mediated increases in extracellular nitric oxide (NO) did not affect cell viability, intracellular ROS increased under NTAPP exposure and induced apoptotic cell death. This effect was dose-dependently reduced following treatment with ROS scavengers. NTAPP induced apoptosis even in doxorubicin-resistant cancer cell lines, demonstrating the feasibility of NTAPP as a potent cancer therapy. Collectively, these results strongly support the potential of NTAPP as a selective anticancer treatment, especially for p53-mutated cancer cells.

## Introduction

Apoptosis is a well-known form of programmed cell death that removes damaged and unwanted cells; it serves as a crucial mechanism to defend tissues and organs from various types of stresses and cell damage [1]. Selective induction of apoptosis in cancer cells is considered an ideal approach for cancer therapy, and many anticancer agents with this mechanism have been developed. However, current approaches still face significant challenges to overcome, including drug resistance, low therapeutic efficiency, and cancer cell selectivity.

The p53 tumor suppressor protein is essential for maintaining genomic stability in mammals. When cells are subjected to various genotoxic and cellular stresses, such as oxidative stress, hypoxia, radiation, or chemotherapeutic drugs, p53 is activated, and its ubiquitin-dependent degradation is blocked, leading to an accumulation of active p53 transcription factor [1]. Activated p53 regulates cell cycle arrest, activation of anti-oxidants and DNA repair, and apoptosis by affecting the expression of its target genes, including the cyclin-dependent kinase (CDK) inhibitor *p21/WAF1* and genes involved in cell death, such as *BAX*, *PUMA*, *NOXA*, and *Fas* [2], [3]. When cells are exposed to oxidative stress, p53 also activates the transcription of sestrin, glutathione peroxidase (GPX), and aldehyde dehydrogenase (ALDH), thus playing a pivotal role in maintaining redox balance and genomic stability under oxidative stress [4], [5]. Mutation of the p53 gene or disruption of pathways that lead to p53 activation have been frequently observed in most types of human cancer [6].

The p53-dependent induction of apoptosis in response to genotoxic damage is an important aspect of tumor suppression. Thus, the loss of p53 in human cancers contributes to aggressive tumor behavior and often promotes resistance of cancer cells to radiation and chemotherapeutic drugs. For example, treatment of p53^+/+^ mouse thymocytes with radiation results in apoptosis, whereas p53^−/−^ thymocytes are resistant. Similarly, p53^+/+^ mouse embryonic fibroblasts transformed by adenoviral E1A protein and Ha-ras oncogene undergo apoptosis in response to γ-irradiation or chemotherapeutic agents, but p53^−/−^ fibroblasts are resistant to both treatments [7]. In addition, some p53 mutations in cancers suppress the function of p73, which induces apoptosis through a p53-independent mechanism [8]. Thus, the common loss of p53 function in cancer cells presents a major limitation for anticancer therapies.

Plasma is described as quasi-neutral mixture of charged particles and radicals in a partially ionized gas. Recently, many studies have attempted to take advantage of the low temperature of non-thermal atmospheric pressure plasmas (NTAPPs) for biomedical applications by the virtue of controllability of plasma chemistry and kinetics [9]–[11]. There are several types of NTAPPs, such as plasma needle, plasma jets, and dielectric barrier discharges (DBDs) [11]. The gas component and the strength and pulse duration of the electric field determine the exact plasma compositions. The study of NTAPPs for clinical applications has recently become a very active research topic; NTAPPs are easily generated in air and can be used without causing thermal damage to cells. The effects of NTAPPs on living tissues include sterilization, wound healing, and cell migration changes (for reviews [10], [12]). The variety of different effects of plasma depends on plasma dosage and their complex chemical compositions.

Previous studies regarding the clinical application of NTAPP for mammalian cells have mainly focused on its effect on cell death [13], [14]. Several possible mechanisms related to NTAPP-mediated cell death were reported, including the decrease of cell adhesion [15], [16]. In particular, several research groups demonstrated that NTAPP induces apoptosis in some cancer cells and may be used as a cancer therapy [17], [18]. There is a growing body of evidence suggesting that reactive oxygen species (ROS) are the major players of NTAPP-induced apoptosis *in vitro* [19]–[22].

ROS are chemically reactive radicals, ions, or molecules containing free oxygen radicals and a byproduct of normal metabolism. Basal levels of ROS activate numerous signaling cascades to promote cell proliferation under normal physiological conditions [23]–[25]. However, excessive ROS levels induce oxidative stress and directly attack DNA, protein, lipids, and other cellular components, ultimately contributing to cell senescence and apoptosis [26], [27].

The first step to develop NTAPP as a potential cancer therapy is to evaluate its selective efficacy toward cancer cells. Here, we demonstrate that NTAPP induces apoptosis in p53-mutated cancer cells but not in primary or stem cells. We also show that NTAPP-mediated increases in intracellular ROS induce apoptotic cell death in a concentration-dependent manner. To further evaluate the feasibility of NTAPP for cancer therapy, we tested NTAPP in doxorubicin-resistant cancer cells and found that it was able to induce apoptosis. Together, these results strongly support the potential of NTAPP as a selective anticancer treatment, especially for p53-mutated cancers and cells that are resistant to existing anticancer drugs.

## Materials and Methods

### Cell culture and NTAPP treatment

Cells used in this study and their sources are summarized in Table 1. HeLa and Hep G2 cells were grown in Dulbecco's modified Eagle's medium (DMEM) supplemented with 10% (v/v) fetal bovine serum (FBS) and 10 mL/L penicillin-streptomycin. G361, HCT116, HCT116 p53^−/−^, HCT15, MES-SA, H1299, HT29, DLD-1, LoVo, doxorubicin-resistant HCT15/CL02 and MES-SA/dx5 cells were grown in RPMI-1640 supplemented with 10% (v/v) FBS and 10 mL/L penicillin-streptomycin. IMR90 and RKO cells were grown in Minimum Essential Medium (MEM) supplemented with 10% (v/v) FBS and 10 mL/L penicillin-streptomycin. YD-9 cells were grown in DMEM∶DMEM/F12 (1∶1) supplemented with 10% (v/v) FBS and 10 mL/L penicillin-streptomycin. Subcutaneous adipose tissue was obtained during elective surgeries with patient consent, as approved by the Samsung Hospital Institutional Review Board (IRB no. KBC11151). Adipose-tissue derived stem cells (ASCs) were isolated from the tissue [28] and grown in DMEM/Ham's F-12 (DMEM/F12) supplemented with 10% (v/v) FBS and 10 mL/L penicillin-streptomycin. All cells were maintained at 37°C under a humidified atmosphere of 5% CO_2_.

**Table 1:** Human cells used in this study.

Name	Cell Type	Source
HeLa	human adenocarcinoma	ATCC CCL-2
IMR90	human lung fibroblast	ATCC CCL-186
ASC	human adipose derived stem cell	IRB No. KBC 11151
YD-9	human oral squamous carcinoma	Korean Cell Line Bank 60502
G-361	human malignant melanoma	ATCC CRL-1424
HCT 116 (p53+/+)	human colorectal carcinoma	ATCC CCL-247
HCT 116-E6 (p53−/−)	human colorectal carcinoma	J. Biol. Chem. (2010) 285 (5); 2986–2995
HT29	human colorectal adenocarcinoma	ATCC HTB-38
H1299	human non-small cell lung cancer	ATCC CRL-5803
RKO	human colorectal carcinoma	ATCC CRL-2577
MES-SA	human uterine sarcoma	ATCC CRL-1976
MES-SA/Dx5	human uterine sarcoma	ATCC CRL-1977
HepG2	human hepatocellular carcinoma	ATCC HB-8065
LoVo	human colorectal adenocarcinoma	ATCC CCL-229
DLD-1	human colorectal adenocarcinoma	ATCC CCL-221
HCT15	human colorectal adenocarcinoma	ATCC CCL-225
HCT15/CL02	human colorectal adenocarcinoma	Bull. Korean Chem. Soc. (2012) 33 (1); 337–339

In order to expose cells to NTAPP, 1×10^5^ cells were seeded in 35-mm cultured plates and incubated for 24 h. Cells were exposed to the indicated dose (5 standard L/min [SLM], 5 V) of NTAPP for 30 s to 1 min each time every h for a maximum of 10 times. When necessary, the NTAPP-exposed cells were further incubated for 15 h before other experiments.

### Western blot analysis

NTAPP-exposed cells were harvested and lysed as described [29]. Histones were extracted with 0.6 N HCl. Samples of total protein (50 µg) or histones were analyzed with anti-caspase-3 (Cell Signaling Technology), anti-poly ADP ribose polymerase (PARP, Cell Signaling Technology), anti-actin (Sigma-Aldrich) sera, and anti-phospho-H2AX (γ-H2AX) sera (Millipore), phospho-p53 (Ser15) (Cell Signaling Technology), PUMA (Cell Signaling Technology) and Bax (Santa Cruz Biotechnology). Horseradish peroxidase-conjugated anti-mouse and anti-rabbit (Jackson Immuno Research) secondary antibodies were used, and the treated membranes were visualized by using the ECL kit (Amersham Biosciences).

### Intracellular ROS detection

HeLa cells (1×10^5^) were seeded on 35-mm dish with glass coverslips and repetitively exposed to 5 V NTAPP for 30 s every h for a total of seven times before intracellular ROS levels were assessed using an ROS detection kit (Invitrogen) according to the manufacturer's protocol. Non-fluorescent carboxyl-H_2_DCFDA can easily permeabilize to living cells and is oxidized by intracellular ROS to emit a bright green fluorescence signal. Tert-butyl hydroperoxide (TBHP), which is known to induce intracellular ROS, was used as a positive control. N-acetyl cysteine (NAC) and sodium pyruvate (SP) were used as intracellular and extracellular ROS scavengers, respectively.

### Extracellular nitrite detection

The production of extracellular nitric oxide (NO) following exposure to NTAPP was determined by measuring the accumulation of nitrite, the stable metabolite of NO, secreted to the culture media. After cells were exposed to NTAPP, 50 µl culture medium was taken, mixed with an equal volume of Griess reagent (1% sulfanilamide, 0.1% naphthylethylenediamine dihydrochloride, and 2% phosphoric acid), and incubated at room temperature for 10 min. The absorbance was measured at 540 nm with a microplate reader (SoftMax Pro 4.0, Molecular Devices) using a calibration curve with a range of 0–100 µM concentrations of NaNO_2_. In the NO scavenger experiment, cells were pretreated with 30, 50, or 100 µM concentrations of carboxy-PTIO (Sigma-Aldrich) that reacts stoichiometrically with NO before they were exposed to NTAPP.

### Transfection of pcDNA-p53-HA

We added 2 µg of the plasmid pcDNA-p53-HA (kindly provided by Dr. J. Song, Yonsei University, Seoul, Korea) in 6 µl linear L-PEI (polyethylenimine), mixed with 150 mM NaCl, and incubated at room temperature for 10 min. The mixture was applied to HT29 cells before overnight incubation.

### Flow cytometry

After 10 repetitive exposures of 5 V NTAPP followed by 15 h incubation, cells were harvested with trypsin-EDTA and fixed with 70% cold ethanol. Cells were treated with 100 µg/ml RNase for 30 min in 37°C and resuspended in 1× binding buffer (10 mM Hepes pH 7.4, 140 mM NaCl, 2.5 mM CaCl_2_). Cells were stained with 0.01 mg/mL propidium iodide (Sigma-Aldrich), or double-stained with 5 µl FITC Annexin V (BD Biosciences) and 5 µl 7AAD (eBioscience) per million cells to detect cells in apoptotic status. Flow cytometry was carried out with FACScalibur (BD biosciences) and CellQuest software.

### Global model analysis of plasma chemistry

Because it is difficult to experimentally measure every species present in NTAPP, plasma engineers developed a global model [30], [31] that calculates spatially averaged chemical species, as well as charged particles, under a given driving condition of external circuits using time-dependent continuity equations for each species. The simulation domain is divided into two regions: the plasma source region and the neutral gas domain without plasmas. The latter region contacts the dish containing the cells. The considered chemical reactions are the same as that in table 2 by Sakiyama *et al.* [31], except for the addition of helium (He) species, which were employed as the buffer gas of the NTAPP device used for this experiment. The total number of species is 68, and the total number of reactions is 826.

The global model used in this study solved continuity equations for heavy particle species and an energy balance equation for electron species. Due to the quasi-neutrality of plasma, electron density can be calculated from the summation of all positive species density and the subtraction of all negative species density. The equations were solved numerically with an in-house code using a fourth-order Runge-Kutta method.

### Statistical analysis

All data are represented as the mean ± standard error of mean (SEM) of at least three independent experiments. We applied *t*-tests to assess statistically significant differences. *p*<0.05 (*) and *p*<0.01 (**) indicates statistical significance compared with the control.

## Results

### Properties of the designed NTAPP device

**Figure 1:**
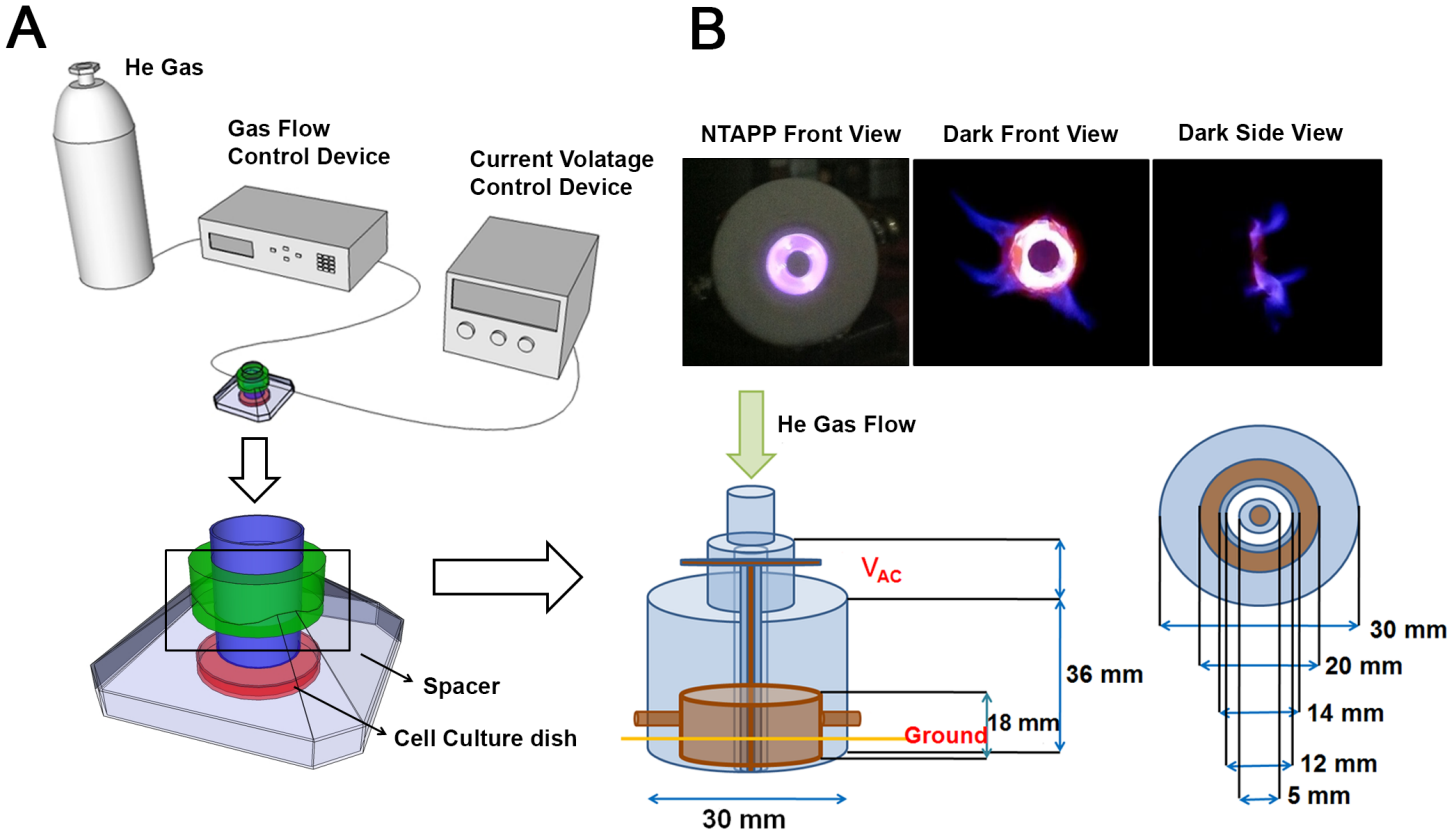
The NTAPP device used in this study. (A) Schematic description of the NTAPP-generating device used to treat living cells. The dielectric material used is Teflon (polytetrafluoroethyle), and the electrode is made of copper. (B) NTAPP generated from the device. The plasma density is on the order of 10^12^/cm^3^, and the total power consumed in the plasma is 1.11 W for a peak to peak voltage of 7 kV with an input DC voltage of 5 V. The amounts of calculated ROS species are shown in Figure S1.

We designed an apparatus that generates NTAPP to expose to live cells. This NTAPP device was designed to have the electric field perpendicular to the direction of gas flow in order to reduce the amount of charged particles through the jet. Thus, the generated ROS diffuse widely, but charged particles are confined within the plasma generation region. The small aspect ratio of the jet area to the plasma volume also results in reduced amount of UV photons from the device. Moreover, the long distance from the device to the cells and the culture media reduce the effects of UV photons that are absorbed by the background gases and liquids very well. Therefore, we expect this device mainly provides ROS from plasma, compared with direct plasma jets that deliver charged particles directly to the targets. Figure 1 shows a schematic picture of the plasma generator used in this experiment. The device is an annular type dielectric barrier discharge (DBD) [32] specified for NTAPP generation under air or other gases when high-voltage sinusoidal wave forms or short duration pulses are applied between two electrodes, at least one of which is insulated. The insulator prevents current build-up between the electrodes, creating electrically safe plasma without substantial gas heating.

As shown in Figure 1, the apparatus is combined with a He gas-feeding system, an AC power supplier, and the DBD device covered with plastic cases to reduce ROS diffusion. He gas flows into the inlet of the device at a rate of 1 to 5 standard liters per minute (SLM), which is controlled by a mass flow controller (MKP, MPR-2000). AC voltage is applied to the inner electrode of the DBD device at a frequency of 11.4 kHz and an applied peak to peak voltage of 7 to 20 kV, which is generated using a transformer circuit composed of rectifier diodes and bipolar power transistors (Ramsey Electronics, PG13 plasma generator kit). The DC input voltage ranges from 5 to 12 V, which is designated as a control parameter to change the applied voltage to the DBD device. For example, the input voltages of 5, 6, and 12 V correspond to peak-to-peak output voltages of 7.5, 8.9, and 18.8 kV, respectively. The total power consumed in the plasma is 14.11 W for the peak to peak voltage of 7 kV with an input DC voltage of 5 V, which is the condition for most of the experimental measurement.

The information regarding the plasma components and composition is indispensable for the reproducibility and mechanism of NTAPP action. However, it is challenging to measure each element generated from the plasma device, especially at atmospheric pressure, because many ROS do not emit light that can be detected in optical emission spectroscopy, and mass spectroscopy is also difficult to use at this pressure. Global modeling is generally used for the analysis of NTAPP reaction kinetics and plasma chemistry under the given operation conditions [30], [31]. Therefore, in order to analyze the components and composition of our NTAPP device, we used global modeling, which is very similar to that of Sakiyama et al. [31], except for the addition of He-related reactions. The results of global modeling with the variation of He mole fraction, as well as humidity, at a fixed input power of 80 W are presented in Figure S1.

For dry air with zero humidity, the reactions related to water are excluded, and thus the total number of species and the total numbers of reactions are reduced to 43 and 454, respectively. As Sakiyama et al. [31] showed two different simulation regions for the discharge layer and neutral gas domain, the simulation domains of discharge region and neutral gas region were considered separately. Figure S1A and S1B are the results of the discharge region, and Figure S1C and S1D are for the neutral gas region contacting the dishes. Figure S1A shows the ROS density inside of the plasma device for different He gas fractions that can be controlled by changing the gas flow rate. Because the threshold energies for interactions with ROS are lower than those with He, the ROS densities are mainly determined by the amount of air, and the most dominant species inside of the discharge region is atomic oxygen due to the electron impact dissociation process of O_2_ and O_3_ [30]. The most abundant species are oxygen atoms, nitric oxide, and ozone when the mixed air fraction is greater than 1%. Figure S1B shows the effect of humidity on ROS density for the mixture of 99% He and 1% air. The considered mole fraction of water vapor is 1%, which corresponds to 30% relative humidity at room temperature. The change in ROS density is not significant with the inclusion of the effect of humidity except for the additional existence of OH, H, H_2_O_2_, HNO_2_, HNO_3_, HO_2_, and H_2_. The ROS generated in the plasma device diffuse out to the air, and they are plotted in Figure S1C after the diffusion time of 0.1 ms that was estimated as the arrival time of the species from the plasma nozzle to the dish. After the diffusion process of the ROS throughout the air, the most dominant species is ozone instead of atomic oxygen, and it easily attaches to O_2_ to generate O_3_. Finally, Figure S1D shows the mole number of ROS arriving at the dish per unit area and unit time. The total mole number can be estimated by multiplying the surface area of the dish and total amount of interaction time. For example, with a dish diameter of 3.5 cm and with operation times of 30 seconds, the total multiplication factor is 289. Therefore, the maximum mole number of ozone delivered to the media is approximately 30 mmol. The transport of ROS inside of liquid media is not well understood and is very hard to calculate, but there are some references for the reaction rates of hydrated electrons and hydroxyl radicals, as well as for transport proton hopping in aqueous solution [31]. If we assume that 10–30% of gaseous ROS are delivered into liquid media, the total mole number inside of the liquid ranges from 3–9 mmol.

### Differential effects of NTAPP on different types of human cells

To determine the optimal exposure conditions of NTAPP to inhibit cancer cell proliferation, the initial experiment assessed the effects of different NTAPP intensities on cell viability in normal and cancerous cells. At first, we applied NTAPP with a range of input voltage (5–10 V) once to the cancer cell line HepG2, the normal embryonic lung fibroblast cell line WI38, and ASCs for 1 min, then the cells were incubated for 24 h before we quantified cell viability using MTT assays. The MTT assay is for assessing cell viability that reflects the number of viable cells present. Increased viability suggests that the treated cells proliferate faster than the control cells. Decreased viability signifies that the treated cells proliferate slower than the control or some of the treated cells undergo cell death. In this experiment, the distance (3 cm) between the NTAPP device and the cells and the gas flow (5 SLM) were fixed.

As shown in Figure S2, while differences in viability were not observed between treated and untreated HepG2 cells, the numbers of WI38-VA13 and ASC cells slightly more increased than the untreated control cells. These results suggest that NTAPP exposure may induce different physiological responses in non-cancerous and cancerous cells. We also verified that the changes in cell viability is due to NTAPP exposure and not by the He gas used for generating NTAPP (Figure S3).

**Figure 2:**
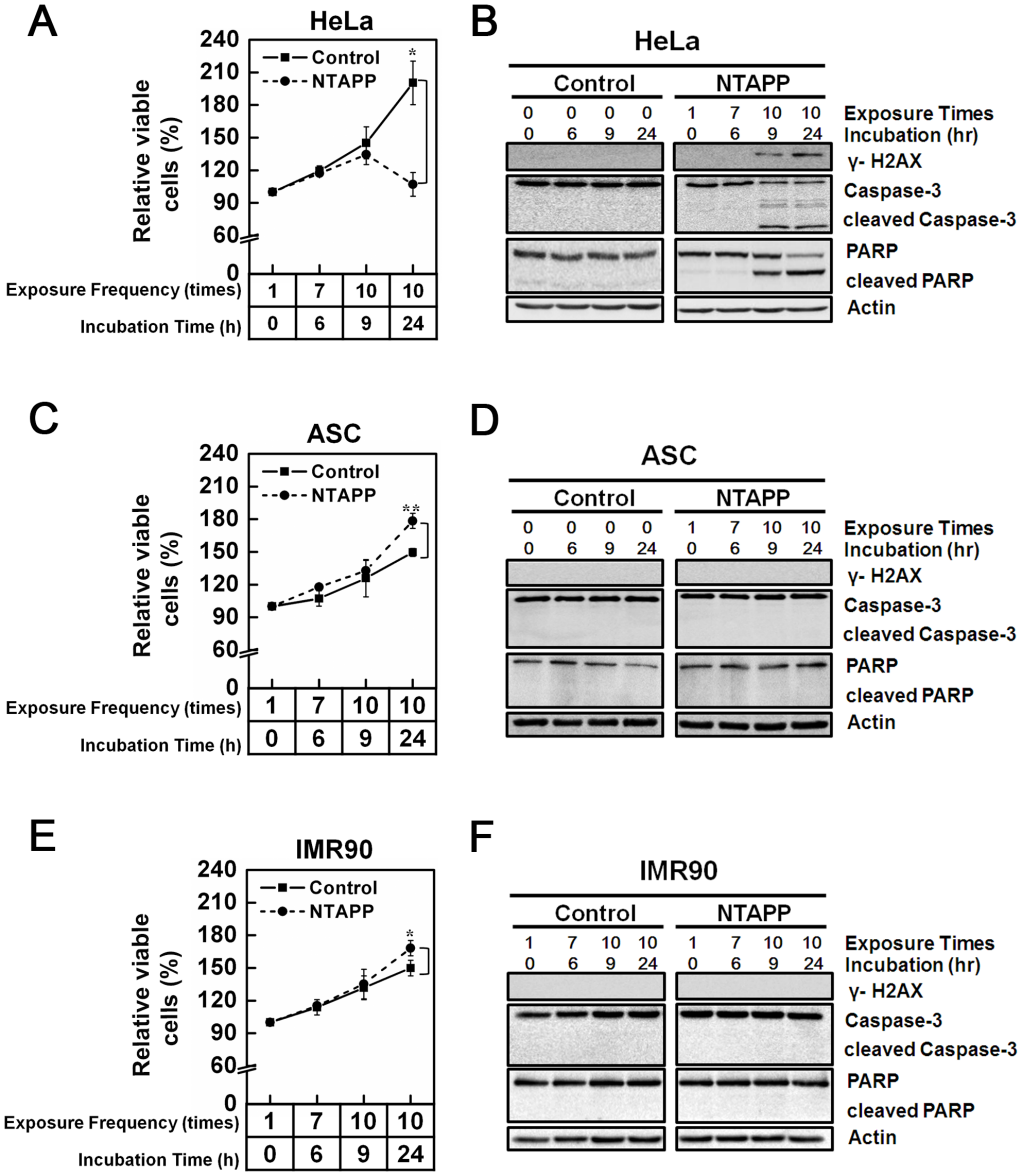
Differential effect of NTAPP on different types of human cells. (A–F) (A, B) HeLa, (C, D) adipose tissue-derived stem cells (ASCs), and (E, F) IMR90 cells were exposed with 5 V input NTAPP for 30 s every h for 10 times, and cell viability was evaluated at each indicated exposure frequency. Incubation time indicates the time after initial exposure to NTAPP. The 24-h incubation sample was prepared with 10 repetitive NTAPP exposures followed by further incubation for 15 h. (A, C, E) The relative percentages of viable cells are shown comparing the initial cell number prior to exposure and incubation as 100%. Viable cells were quantified with MTT assays, and data are shown as mean ± SEM from three independent experiments. *p*<0.05 (*) and *p*<0.01 (**) indicate significant differences compared with the control condition. (B, D, F) For the same NTAPP-exposed samples of (A), (C), (E), respectively, γ-H2AX, caspase-3, cleaved caspase-3, PARP, and cleaved PARP were assessed by western blot analyses. Actin is shown as a loading control.

We next tried to deduce the proper NTAPP conditions to block proliferation in cancer cells but not in normal cells. When HeLa cells were exposed to 5 V NTAPP for 30 s every h for a maximum 9 h (10 repetitive NTAPP exposures), as shown in Figure 2A, the relative percentage of viable cells was slightly less increased in exposed cells (134%) compared to unexposed control cells (145%). In addition, the difference in the relative viable cells between NTAPP-treated and untreated HeLa cells became more pronounced when cells were further incubated for 15 h after 10 NTAPP exposures: the relative percentage of viable cells was only 106% in cells exposed to 10 repetitive NTAPP with 15 h further incubation, compared to 200% in untreated control (Figure 2A). Interestingly, when we applied the same conditions of NTAPP to primary fibroblast IMR90 cells and ASCs, the relative number of cells was augmented in NTAPP-exposed cells compared to untreated control (Figure 2C and E). The relative percentages of viable cells in ASC and IMR90 cells that were exposed to NTAPP 10 times and further incubated for 15 h were 178% and 168%, respectively, whereas that of untreated cells was approximately 150% (Figure 2C and E). These observations strongly suggest that we employed the right NTAPP exposure conditions (repetitive exposure of 5 V input NTAPP with further incubation) to selectively inhibit HeLa cell proliferation and activates proliferation in IMR90 cells and ASCs.

We then examined whether decreased HeLa cell proliferation following repetitive NTAPP exposure was due to apoptosis by monitoring the cleavage of caspase-3 and its downstream effector PARP, both of which are activated by apoptosis. When exposed to 10 repetitive 5 V NTAPP for 30 sec each, cleaved caspase-3 and PARP were detected in HeLa cells but not in IMR90 or ASC cells (Figure 2B). Consistent with the relative cell numbers shown (Figure 2A, C, and E), cleaved caspase-3 and PARP were significantly increased by the additional 15 h incubation in HeLa cells but were not observed in ASCs or IMR90 cells (Figure 2B, D, and F). These results demonstrate that NTAPP treatment induces apoptosis in HeLa cells but not in normal ASC or IMR90 cells. We also confirmed that repetitive NTAPP exposure induces apoptosis in HeLa cells using flow cytometry with Annexin-V/7AAD staining (Figure S4).

Considering that genomic instability due to DNA damage is a major cause of apoptosis, we investigated whether NTAPP exposure induced DNA damage in apoptotic HeLa cells. The phosphorylation of histone H2AX is one of the earliest cellular events that occur in response to DNA double-strand breaks [7]. While cancer cells, including HeLa, usually show low basal γ-H2AX expression without any stress, it was significantly increased in NTAPP-exposed HeLa cells (Figure 2B). Not surprisingly, no γ-H2AX expression was detected in ASCs or primary IMR90 cells (Figure 2D and F). This observation indicates that NTAPP exposure selectively induces DNA damage that leads to HeLa cell apoptosis.

### Anti-proliferative effect of NTAPP on melanoma and oral carcinoma cells

**Figure 3:**
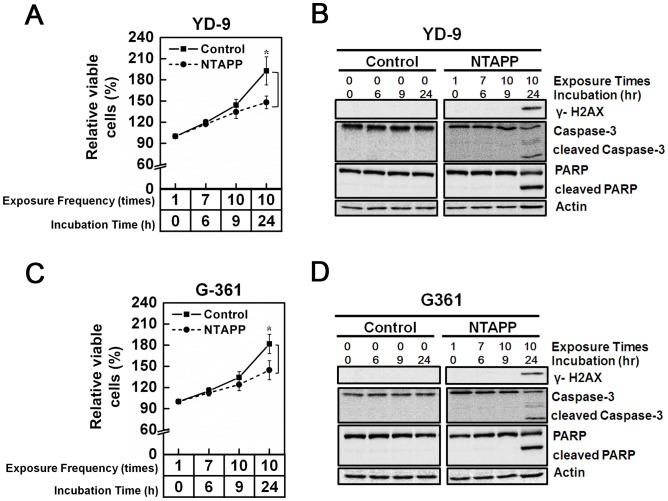
Anti-proliferative effect of NTAPP on melanoma and oral carcinoma cells. (A–D) (A, B) Oral squamous carcinoma YD-9 and (C, D) melanoma G361 cells were exposed with 5 V input NTAPP for 30 s every h for 10 times, and cell viability was evaluated at each indicated exposure frequency. Incubation time indicates the time after initial exposure to NTAPP. The 24-h incubation sample was prepared with 10 repetitive NTAPP exposures followed by further incubation for 15 h. (A, C) The relative percentages of viable cells are shown comparing the initial cell number prior to exposure and incubation as 100%. Viable cells were quantified with MTT assays, and data are shown as mean ± SEM from three independent experiments. *p*<0.05 (*) and *p*<0.01 (**) indicate significant differences compared with the control condition. (B, D) For the same NTAPP-exposed samples of (A) and (C), respectively γ-H2AX, caspase-3, cleaved caspase-3, PARP, and cleaved PARP were assessed by western blot analyses. Actin is shown as a loading control.

Because the exposure of NTAPP selectively induces apoptosis in HeLa cells but not in primary IMR90 or ASCs, we examined its effect on other types of cancer cells, including melanoma G361 and oral squamous carcinoma YD-9 cells, which are derived from cancers found in the surface of the body where plasma treatment could be readily applicable. As shown in Figure 3A and C, 10 repetitive exposures of 5 V NTAPP followed by 15 h incubation had an anti-proliferative effect on G-361 and YD-9 cells compared with untreated control cells. NTAPP exposure also induced γ-H2AX expression by DNA damage and apoptosis in G-361 and YD-9 cells (Figure 3B and D). These results demonstrate that as in HeLa cells, NTAPP exposure leads to substantial apoptotic cell death in melanoma and oral squamous carcinoma cells, suggesting the potential of plasma application to skin and oral cancers.

### Highly preferential anti-proliferative effect of NTAPP on p53-deficient cancer cells

Under the same NTAPP conditions, its anti-proliferative effect was greater in HeLa cells than in G-361 and YD-9 cells (Figure 2A and 3), although all cell types eventually underwent apoptosis. γ-H2AX phosphorylation and apoptosis were observed in HeLa cells when 10 repetitive NATPP stimulations were applied, but this was only observed in G-361 and YD-9 cells when cells were further incubated for 15 h after NTAPP exposure. HeLa, G-361, and YD-9 are all cancer cells, but only HeLa cells lack functional p53 [33]–[35]. Considering that NTAPP induces apoptosis through DNA damage and that p53 is the key tumor suppressor in response to genotoxic stresses, we postulated whether the presence or absence of functional p53 in HeLa, YD-9, and G361 cells makes the difference in the anti-proliferative effect of NTAPP.

**Figure 4:**
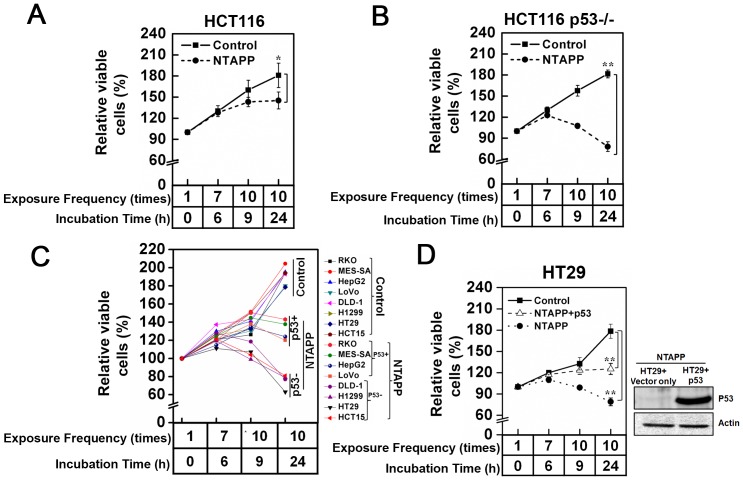
Highly preferential anti-proliferative effect of NTAPP on cancer cells without functional p53. (A–D) Indicated cancer cell lines were exposed with 5 V input NTAPP for 30 s every h for 10 times, and cell viability was evaluated at each indicated exposure frequency. Incubation time indicates the time after the initial NTAPP exposure. The 24 h incubation was prepared with 10 repetitive NTAPP exposures followed by further incubation for 15 h. The relative percentages of viable cells are shown comparing the initial cell number prior to exposure and incubation as 100%. Viable cells were quantified with MTT assays, and data are shown as mean ± SEM from three independent experiments. *p*<0.05 (*) and *p*<0.01 (**) indicate significant differences compared with the control condition. (A, B) After NTAPP exposure, the relative percentage of viable cells were plotted for (A) HCT116 (p53+/+) and (B) HCT116-E6 (p53−/−), both of which have the same genetic background except for the (A) presence and (B) absence of functional p53. (C) The relative percentages of viable cells were plotted together after the same NTAPP exposures in p53-proficient cells (RKO, MES-SA, HepG2, G361, LoVo) and p53-deficient cells (DLD-1, H1299, HT29, HCT115). (D) p53-deficient HT29 cells were transfected with pcDNA-p53-HA, and the expression of p53 in HT29 was verified by a western blot shown on the right side with actin as a loading control. The relative percentages of viable cells in HT29 and p53-transfected HT29 cells were plotted after the same NTAPP exposures.

In order to demonstrate the relationship between the function of p53 and the sensitivity of cancer cells to NTAPP, we compared the anti-proliferative effect of NTAPP in colorectal cancer cell lines HCT116 (p53^+/+^) and HCT116-E6 (p53^−/−^), both of which have the exact same genetic background except for functional p53. When HCT116 (p53^+/+^) and HCT116-E6 (p53^−/−^) received 10 repetitive 5 V NTAPP stimulations followed by 15 h further incubation, the same condition used in the previous NTAPP treatment, the relative percentage of viable cells were decreased both in HCT116 (p53^+/+^) and HCT116-E6 (p53^−/−^) compared with untreated control cells (Figure 4A and B). Interestingly, HCT116-E6 (p53^−/−^) cells showed hypersensitivity to NTAPP compared with HCT116 (p53^+/+^); the relative percentage of viable cells was only 80% in HCT116-E6 and 140% in HCT116, compared to 180% in both untreated control groups (Figure 4A and B). These results are consistent with the effect of NTAPP observed for HeLa, YD-9, and G361 cells and strongly suggest that cancer cells without functional p53 are significantly more sensitive to NTAPP than cells that with functional p53.

Because more than 50% human cancer cells have p53 gene mutations that cause defects in the function of p53 and the lack of functional p53 usually allows cancer cells to resist γ-irradiation or chemotherapeutic agents [7], the highly preferential anti-proliferation effect of NTAPP on cancer cells without functional p53 is of great interest. To confirm the highly preferential anti-proliferative effect of NTAPP on cancer cells without functional p53, we examined the relative percentage of viable cells in various cancer cell types with or without the function of p53 after NTAPP treatment. The number of viable cells were decreased by NTAPP exposure in both p53-positive and -negative cells, compared with the cells that were not exposed to NTAPP (Figure 4C). As expected, the anti-proliferative effect of NTAPP was much more efficient in cancer cells without functional p53 (DLD-1, H1299, HT29, and HCT15) than in cancer cells with wild-type p53 (LoVo, MES-SA, HepG2, and RKO): the relative percentage of viable cells was around 60–80% in p53-negative cancer cells while it was approximately 120–140% in p53-positive cancer cells (Figure 4C). These results further demonstrated that the anti-proliferative effect of NTAPP is highly selective to p53-negative cancer cells.

In order to further verify the reverse correlation between the function of p53 and the anti-proliferative effect of NTAPP in cancer cells, we compared the number of viable cells after NTAPP exposure in p53-negative HT29 cells transfected with p53-expressing vector and vector-only control. The expression of transfected p53 in HT29 was verified by western blot (Figure 4D). The HT29 cancer cell line does not contain functional p53, and its viability decreased sharply following NTAPP treatment (Figure 4C). However, the relative number of viable cells increased in HT29 cells expressing wild-type p53 to the level observed in other p53-proficient cancer cells (Figure 4C), while there was no change in viability in control HT29 cells transfected only with the vector (Figure 4D). These results demonstrate that cell viability was recovered in p53-transfected cancer cells. In short, these observations confirm that the anti-proliferative effect of NTAPP is highly preferential toward p53-deficient cancer cells and strongly suggest that NTAPP would be an efficient therapy in cancer cells resistant to γ-irradiation or chemotherapeutic agents due to the lack of functional p53.

### p53 activation and G1 cell cycle delay by NTAPP in p53-proficient cancer cells

**Figure 5:**
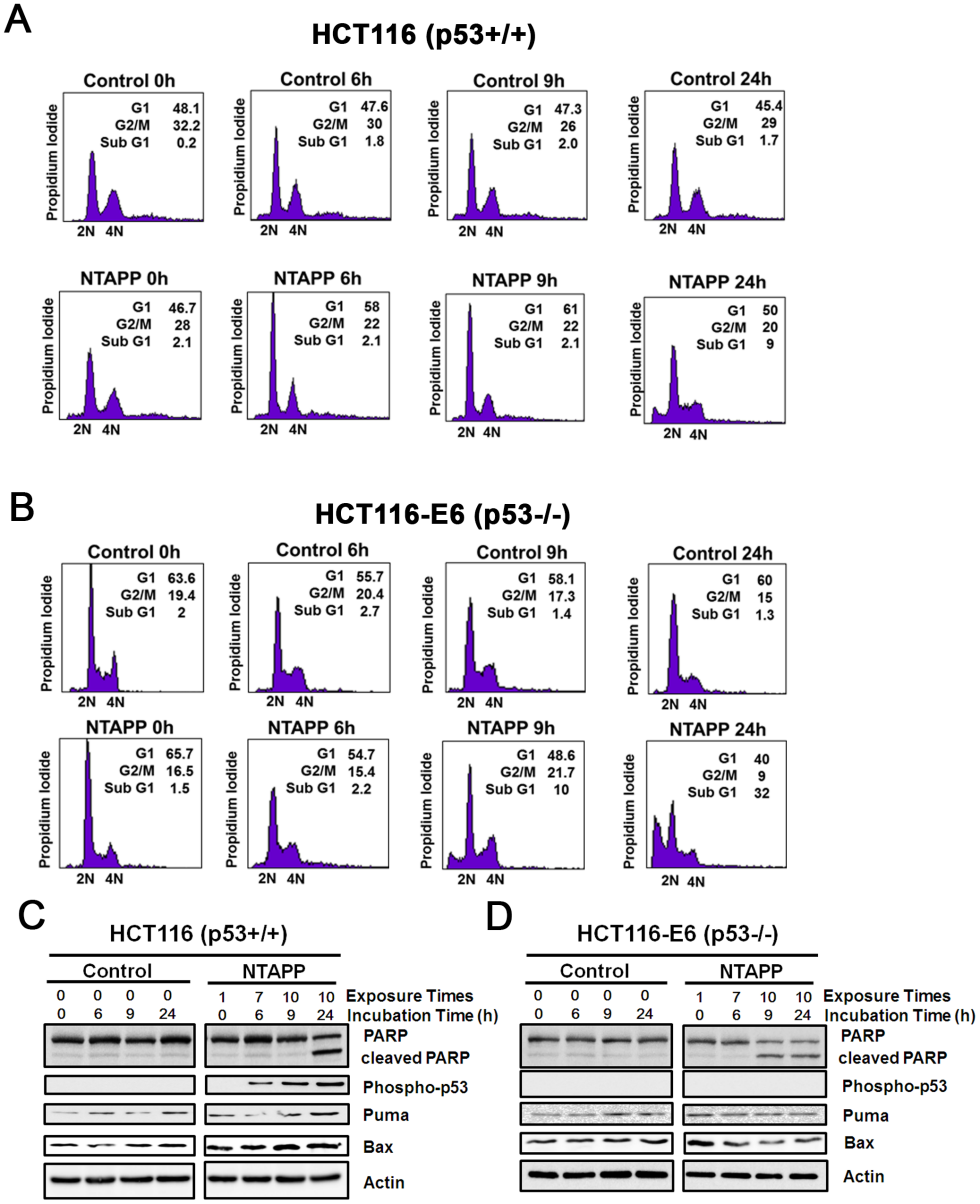
p53 activation and G1 cell cycle delay by NTAPP in p53-proficient cancer cells. (A, B) HCT116 (p53+/+) and (B) HCT116-E6 (p53−/−) cells were exposed with 5 V input NTAPP for 30 s every h for maximum 10 times, and DNA content was evaluated by flow cytometry after propidium iodide staining at each indicated incubation time, which indicates the time after initial exposure to NTAPP. The 24 h incubation sample was prepared with 10 repetitive NTAPP exposures followed by further incubation for 15 h. (C, D) In the same NTAPP-exposed samples of (A, B), PARP, cleaved PARP, Ser 15 phophorylated p53, Puma and BAX were assessed respectively by western blot analyses. Actin is shown as a loading control.

Our results showed that p53-deficient cancer cells are highly more sensitive to NTAPP than p53-proficient cancer cells. Thus, we examined whether p53 plays an important role to control cell cycle and apoptosis in response to NTAPP exposure in cancer cells. The cell cycle progression and the expression of p53 downstream targets are compared in colorectal cancer cell lines HCT116 (p53^+/+^) and HCT116-E6 (p53^−/−^), both of which have the exact same genetic background except for functional p53. Because HCT116 and HCT116-E6 DNA cancer cells have defects in DNA mismatch repair system, even without NTAPP exposure, about 48% of HCT116 and 63% of HCT116-E6 cells contained the 2N DNA content, suggesting a delay in G1/S (Figure 5A and B). In HCT116, the cells in G1 increased to 61% of population by 10 NTAPP exposures (9 h), suggesting the G1 delay of cell cycle (Figure 5A). Further incubation of these NTAPP-exposed HCT116 cells for 15 h (24 h) increased the sub G1 population, showing that some cells had undergone apoptosis (Figure 5A). On the other hand, 10 NTAPP exposures did not increased the cell with 2N DNA content but increased sub G1 population quickly in HCT116-E6 (9 h), demonstrating that these NTAPP-exposed cells undergo apoptosis without G1 cell cycle delay (Figure 5B). These results suggest that NTAPP exposures lead to G1 cell cycle delay in a p53-dependent manner prior to apoptosis in p53-proficient cells.

We then examined the activation of p53 and the expression of its downstream targets, PUMA and Bax, by NTAPP exposure in HCT116 cells [3]. We measured the activation of p53 by detecting the phosphorylation of Ser 15 in p53 [36]. Phophorylated p53 as well as the expression of PUMA and Bax has increased with NTAPP exposures in p53-proficient HCT116 cells (Figure 5C). Meanwhile, apoptosis progressed rapidly and cleaved PARP was detected early in HCT116-E6 where no p53 activation has been detected (Figure 5D). However, the expression of PUMA and Bax was not changed in NTAPP-exposed HCT116-E6 cells (Figure 5D). Altogether, these observations show that NTAPP exposures induce DNA DSB and activate p53 to delay the cell cycle at G1 and to lead to apoptosis in p53-proficient cells, while NTAPP expedites p53-independent apoptosis in p53-deficient cells.

### Anti-proliferative roles of ROS generated by NTAPP

Previous studies by several research groups suggest that ROS are major players in the cell response induced by plasma [37]–[41]. So far, we have demonstrated that NTAPP has a selective anti-proliferative effect in cancer cells, especially in p53-deficient cancer cells, which is achieved by inducing DNA DSBs and apoptosis. In addition, the plasma device used in this study is expected to generate various ROS (Figure S1). Thus, we wanted to know whether the selective anti-proliferative effect of NTAPP shown in this study is also due to ROS, and if so, whether the intra- or extracellular ROS mediate the biological effect of NTAPP. For this purpose, we used an anti-oxidant and a ROS scavenger: NAC is a well-known thiol antioxidant, and SP is a well-known scavenger of hydrogen peroxide (H2O2) [42].

**Figure 6:**
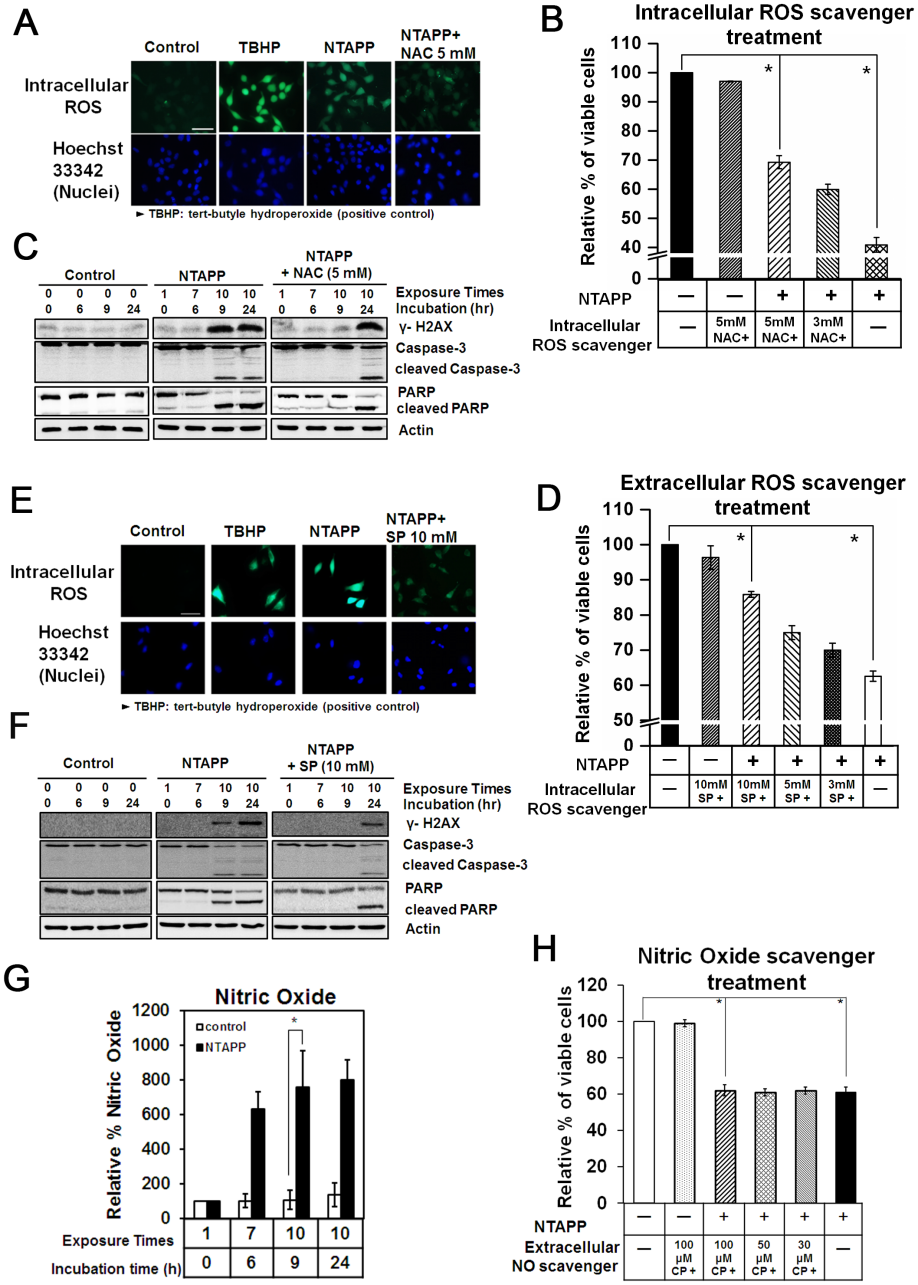
Anti-proliferative roles of ROS generated by NTAPP exposures. (A) HeLa cells pretreated with 5 mM N-acetyl cysteine (NAC), only with culture medium (a negative control), or 100 µM tert-butyl hydroperoxide (TBHP, a positive control of ROS generation) were exposed with 5 V input NTAPP for 30 s every h for seven times. The intracellular ROS generated was monitored with fluorescent microscopy. Nuclei were stained with Hoechst 33342. Scale bar, 50 µm. (B) HeLa cells pretreated with different concentrations of NAC (untreated, 3 mM, 5 mM) were exposed with 5 V input NTAPP for 30 s every h 10 times and further incubated for 15 h. The relative percentages of viable cells are shown comparing the initial cell number prior to exposure and incubation as 100%. Viable cells were quantified with MTT assays, and data are shown as mean ± SEM from three independent experiments. *p*<0.05 (*) indicates a significant difference compared with the control. (C) For the same NTAPP-exposed samples of cells in (B) that were untreated or pretreated with 5 mM NAC, γ-H2AX, caspase-3, cleaved caspase-3, PARP, and cleaved PARP were assessed by western analyses. Actin is shown as a loading control. (D) HeLa cells pretreated with 10 mM sodium pyruvate (SP), only with culture medium (a negative control), or 100 µM TBHP were exposed with 5 V input NTAPP for 30 s at every h seven times. The intracellular ROS generated was monitored by detection with CM-H2DCFDA reagent using fluorescent microscopy. Nuclei were stained with Hoechst 33342. Scale bar, 50 µm. (E) HeLa cells pretreated with different concentrations of SP (0, 3, 5, 10 mM) were exposed with 5 V input NTAPP for 30 s every h 10 times and further incubated for 15 h. The relative percentages of viable cells are shown comparing the initial cell number prior to exposure and incubation as 100%. Viable cells were quantified with MTT assays, and data are shown as mean ± SEM from three independent experiments. *p*<0.05 (*) indicates significant difference compared with control. (F) For the same NTAPP-exposed samples in (E) untreated or pretreated with 10 mM SP, γ-H2AX, caspase-3, cleaved caspase-3, PARP, and cleaved PARP were assessed by western analyses. Actin is shown as a loading control. (G) The NO concentrations in the media were measured with Griess tests after NTAPP exposures. Data are shown as the mean ± SEM from three independent experiments. *p*<0.05 (*) indicates significant difference compared with control. (H) HeLa cells were pretreated with different concentrations of a NO scavenger, carboxy-PTIO (0, 30, 50, 100 µM), exposed to NTAPP 10 times, and further incubated for 15 h. Cell viability was quantified with MTT assay, and the relative percentages of viable cells were plotted comparing the levels in untreated cells. Data are shown as mean ± SEM from three independent experiments. *p*<0.05 (*) indicates significant difference compared with control.

First, we monitored intracellular ROS levels in HeLa cells exposed to NTAPP using the oxidation-sensitive fluorescent dye, DCFDA. Cells treated with TBHP served as a positive control for intracellular ROS [43]. As shown in Figure 6A, intracellular ROS rapidly accumulated in HeLa cells after 7 repetitive 30 s exposures to 5 V NTAPP (exposure at each h), when compared with untreated control. In contrast, intracellular ROS accumulation was efficiently blocked in HeLa cells exposed to the same NTAPP conditions in the presence of 5 mM NAC (Figure 6A). Before using NAC, we assessed the cytotoxicity of NAC and verified that 5 mM NAC did not affect cell viability, as shown in Figure S5.

To examine the role of intracellular ROS in the anti-proliferative effect of NTAPP, we assessed the relative cell viability of HeLa cells with or without NTAPP treatment in the presence of different concentrations of NAC (3 and 5 mM). HeLa cell viability was sharply decreased by NTAPP exposure, but it was recovered by the presence of NAC in a concentration-dependent manner, as shown in Figure 6B: the relative percentage of viable cells to NTAPP-untreated cells (100%) was 70% in 5 mM NAC, 60% in 3 mM NAC, and 40% in the absence of NAC. These results demonstrate that the intracellular ROS generated by NTAPP play a major role in the anti-proliferative effect of NTAPP. Consistent with this, when we compared DNA damage and apoptosis in HeLa cells exposed to NTAPP in the presence and absence of 5 mM NAC, γ-H2AX phosphorylation by DNA damage and apoptosis was delayed in the presence of NAC (Figure 6C). The anti-proliferative effect of intracellular ROS generated from NTAPP was further confirmed with an intracellular ROS scavenger, SP. When HeLa cells were exposed to NTAPP in the presence of SP, intracellular ROS were reduced, and the relative number of viable cells was increased in a concentration-dependent way compared with NTAPP-exposed cells that were not treated with SP (Figure 6D and E). The relative percentage of viable cells to the untreated controls (100%) was 85% with 10 mM SP, 75% in 5 mM, and 70% in 3 mM, compared to only 60% in HeLa cells without SP (Figure 6D). γ-H2AX phosphorylation by DNA damage and apoptosis was also delayed and reduced in the presence of SP as in NAC (Figure 6F). Collectively, these observations strongly support the notion that intracellular ROS generated by NTAPP play key roles in inducing DNA damage and apoptosis to block cancer cell proliferation.

Exposure to NTAPP increases both intra- and extracellular ROS. We wanted to determine whether the plasma-mediated increase in extracellular ROS directly influenced the anti-proliferative effect of NTAPP in cancer cells. We are especially interested in whether NO is easily generated when plasma meets N2 and O2 in the air; NO is known to be an important cellular messenger molecule involved in many physiological and signaling pathways in mammals [44], [45]. Thus, we detected NO in the media with Griess reagent assays [46] after cells were exposed to NTAPP. As shown in Figure 6G, after NTAPP treatment, the concentration of NO in the media was increased more than 7–8 fold compared with untreated control, and the increased NO persisted in the media even with 15 h further incubation after NTAPP exposure (Figure 6G). To assess the anti-proliferative effect of NO generated in the media by NTAPP, we used a NO scavenger, 2-(4-carboxyphenyl)-4,4,5,5-tetramethylimidazoline-1-oxyl 3-oxide (carboxy-PTIO) [47]. The relative viability of HeLa cells was examined after cells were treated or untreated with NTAPP in the presence of different concentrations of carboxy-PTIO in the media (30, 50, or 100 µM). The relative percentage of viable cells was decreased to about 60% by NTAPP exposure compared with untreated cells (100%). However, there was no difference in viability by NTAPP exposure regardless of the addition of carboxy-PTIO (Figure 6H), suggesting that the extracellular NO generated by NTAPP does not influence the anti-proliferative effect of NTAPP.

Taken together, the data indicate that intracellular ROS generated by NTAPP exposures play an important role in inducing apoptotic and anti-proliferative effects in cancer cells, but extracellular NO does not. In addition, as you see in Figure 6, the calculated value of ROS in Figure S1 is relevant to the amount of ROS scavenger used to control intracellular ROS, supporting the model used to predict the plasma chemistry in this study.

### Anti-proliferative effect of NTAPP on doxorubicin-resistant cancer cells

**Figure 7:**
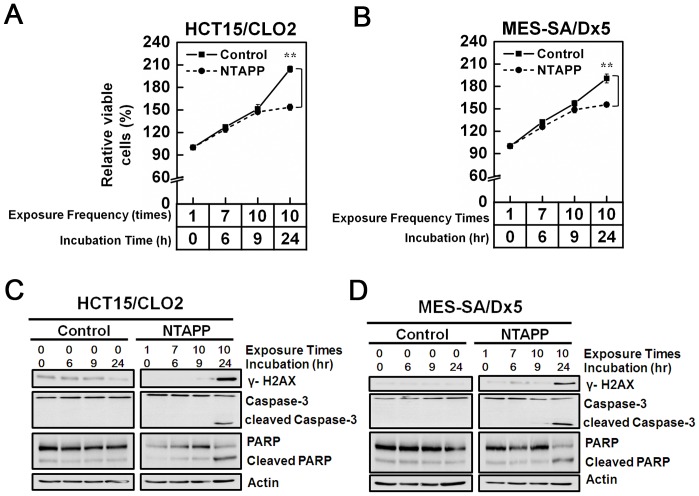
Anti-proliferative effect of NTAPP on doxorubicin-resistant cancer cells. (A–D) (A, B) HCT15/CLO2 (C, D) and MES-SA/dx5 cells were exposed with 5 V input NTAPP for 30 s every h 10 times, and cell viability was evaluated at each indicated exposure frequency. Incubation time indicates the time after the initial NTAPP exposure. The 24-h incubation was prepared with 10 repetitive exposures of NTAPP and further incubation for 15 h. (A, C) The relative percentage of viable cells is shown compared with the initial cell number prior to the exposure and incubation as 100%. Viable cells were quantified with MTT assays, and data are shown as the mean ± SEM from three independent experiments. *p*<0.01 (**) indicates a significant difference compared with the control. (B, D) For the same NTAPP-exposed samples of (A) and (C), respectively, γ-H2AX, caspase-3, cleaved caspase-3, PARP, and cleaved PARP were assessed by western blot analyses. Actin is shown as a loading control for western blot.

The main hurdle in chemotherapy is overcoming the drug-resistance of cancer cells. Because we found that NTAPP has a selective anti-proliferative effect on cancer cells and may be a potential anti-cancer therapy, we also tested whether the NTAPP exposure inhibits the proliferation of anticancer drug-resistant cancer cells. We used two different doxorubicin-resistant cancer cell lines, human colon carcinoma-derived HCT15/CL02 and human uterus sarcoma-originated MES-SA/dx5 cells [48], [49]. Doxorubicin is a commonly used chemotherapy in the treatment of a wide range of cancers including hematological malignancies, many types of carcinoma, and soft tissue sarcomas. When exposed with 10 repetitive 30 s 5 V input NTAPP (one exposure at each hour) and further incubated for 15 h, the same exposure condition used for other cancer cells in this study, the relative percentage of viable cells were significantly decreased in both in HCT15/CLO2 and MES-SA/DX5 cells (less than 150%), compared with untreated cells (200%) (Figure 7A and B). The NTAPP exposure induced γ-H2AX phosphorylation by DNA damage and apoptosis in HCT15/CLO2 and MES-SA/DX5 cells as well (Figure 7C and D). These data showed that NTAPP also induces apoptosis and inhibits the proliferation of doxorubicin-resistant cancer cells, strongly suggesting that NTAPP has a potential to be an effective anticancer therapy even to cancer cells that develop resistance to known chemotherapies.

## Discussion

NTAPP has been suggested as a new cancer therapy. In order to develop NTAPP as a potential cancer therapy, its selectivity to cancer cells must first be documented. In this study, we designed an NTAPP-generating apparatus and determined the right exposure conditions to elicit a selective anti-proliferative effect on cancer cells but not primary fibroblast IMR90 or ASCs. Our results verify that the anti-proliferative effect of NTAPP is highly efficacious and preferential toward p53-deficient cancer cells.

More than 50% of human tumors have a mutation that affects the function of the tumor suppressor p53 [50]. Inheriting a mutated p53 allele increases cancer susceptibility and reduces sensitivity to anti-cancer therapies. Because cells with defective p53 are relatively resistant to apoptosis and, thus to chemotherapeutic agents, a new treatment that preferentially induces apoptosis in p53-defective cancer cells would be an effective anticancer therapy. In this regard, our finding of a NTAPP treatment that exerts a highly preferential anti-proliferative effect on p53-deficient cancer cells suggests that NTAPP has great potential as a new anticancer therapy.

Our results demonstrate the merit of NTAPP as a potential anticancer therapy in that NTAPP has an anti-proliferative effect on doxorubicin-resistant cancer cells. This quality of NTAPP is important as chemotherapeutic drug-resistance of cancer cells has been a big obstacle for cancer treatment efficacy. One limitation of using NTAPP as a cancer treatment would be the accessibility of NTAPP to cancer cells, since plasma cannot penetrate the body. Thus, NTAPP may first be applied to skin or oral cancers as shown in Figure 3.

One question is why cancer cells, and especially p53-deficient cancer cells, are more sensitive to NTAPP. We and others showed that the anti-proliferative, apoptotic effect of NTAPP is due to increased intracellular ROS (Figure 6). ROS activates p53 to delay cell cycle and apoptosis in p53-proficient cells [51]–[53]. We showed that the hypersensitivity of p53-deficient cancer cells to NTAPP is correlated with the lack of p53-dependent cell cycle delay at G1 (Figure 5). Compelling evidence indicates that cancer cells, especially p53-defective cells, have higher levels of endogenous ROS than normal cells due to oncogene hyperactivation and aberrant metabolism [52], [54], [55]. Thus, cancer cells have to survive in higher intrinsic oxidative stress environments and become more vulnerable to damage by ROS-generating agents [56]–[58]. Some research groups have attempted to modulate redox homeostasis to selectively induce apoptosis in cancer cells without significant damage to normal cells [54], [59], [60]. Thus, the high selectivity of NTAPP toward cancer cells observed here might be attributable to differential sensitivity of cancer cells versus primary and stem cells to increased intracellular ROS by NTAPP. In fact, we observed that cell proliferation is noticeably increased in ASCs exposed to NTAPP (Figure 2C). The preferential anti-proliferative effect of NTAPP toward p53-deficient cells might also be due to defective redox homeostasis in p53-defective cells because wild-type p53 regulates the up-regulation of several antioxidant genes [4], [5]. Alternatively, Volotskova et al. hypothesized that the increased sensitivity of cancer cells to plasma treatment is due to differences in the distribution of cancer cells and normal cells within the cell cycle [20].

It is not yet clear what particular component of plasma induces the increase of intracellular ROS. NTAPP could directly and/or indirectly cause changes in ROS components and composition. The physiological effect of NTAPP on cells could be due to a combination of the interactions between various electromagnetic radiations with ions, electrons, and reactive chemical species in the cell. In order to develop NTAPP as an effective anticancer therapy, further studies into the chemistry and kinetics of plasma are necessary to understand the physiological mechanism of NTAPP and to increase its reproducibility.

## Supporting Information

Figure S1 **Characterization of NTAPP components and composition.** The horizontal axis shows the species names, and the vertical axis shows the density, flux, and mole number. (A) ROS density inside the plasma device without water vapor for the variation of partial fraction of He gas. (B) The comparison of the effect of water vapor with 1% air added in 99% helium. The mole fraction of water vapor is 1%, which corresponds to 30% of relative humidity at room temperature. (C) The amount of ROS diffused into the air after 0.1 ms to reach the surface of dishes containing cells. (D) The mole number of ROS arriving at the dish per unit time and unit area.
(TIF)

Figure S2 **Establishment of optimal exposure conditions for NTAPP.** HepG2, WI38-VA13, and adipose tissue-derived stem cells were once exposed for 1 min to NTAPP of various input voltages with 5 SLM (input gas flow ratio to generate NTAPP: standard liter per minute) and 3 cm (the distance between NTAPP to the cell surface) condition. Cells were further incubated for 24 h after NTAPP exposure, and viable cells were quantified with MTT assays. The relative percentages of viable cells were plotted compared with the untreated cells. Data are shown as the mean ± SEM from three independent experiments.
(TIF)

Figure S3 **He gas used for NTAPP generation does not affect cell viability.** HeLa cells were only exposed to 5 slm He gas for 30 s every h 10 times, and the viable cells were evaluated by MTT assays. The relative percentages of viable cells were plotted compared with the initial cells prior to NTAPP exposure and incubation. Data are shown as the mean ± SEM from three independent experiments.
(TIF)

Figure S4 **Reduced viability by NTAPP in HeLa cells results from apoptosis.** HeLa cells were exposed with 5 V input NTAPP for 30 s every h 10 times, and the induction of apoptosis was determined by flow cytometric analysis with Annexin V-FITC and 7AAD-staining at each indicated exposure frequency. Incubation time indicates the time after the initial NTAPP exposure. The 24 h incubation was prepared with 10 repetitive exposures of NTAPP and further incubation for 15 h. Cells in the lower right quadrant indicate Annexin-positive, early apoptotic cells. The cells in the upper right quadrant indicate Annexin-positive/7AAD-positive, late apoptotic cells.
(TIF)

Figure S5 **The cytotoxicity of N-acetyl cysteine on HeLa cells.** To document the cytotoxicity of the ROS scavenger N-acetyl cysteine (NAC), HeLa cells were incubated in the presence of different concentrations (0, 3, 5, 10 mM) of NAC for 12 h, and viable cells were quantified using MTT assays. The relative percentages of viable cells were plotted compared with the untreated cells. Data are shown as the mean ± SEM from three independent experiments.
(TIF)
