# Supplementary figures and images for: Non-Thermal Atmospheric Pressure Plasma Preferentially Induces Apoptosis in p53-Mutated Cancer Cells by Activating ROS Stress-Response Pathways
Source: PLoS One. 2014 Apr 23;9(4):e91947. doi: 10.1371/journal.pone.0091947 (PMC3997341; doi:10.1371/journal.pone.0091947)

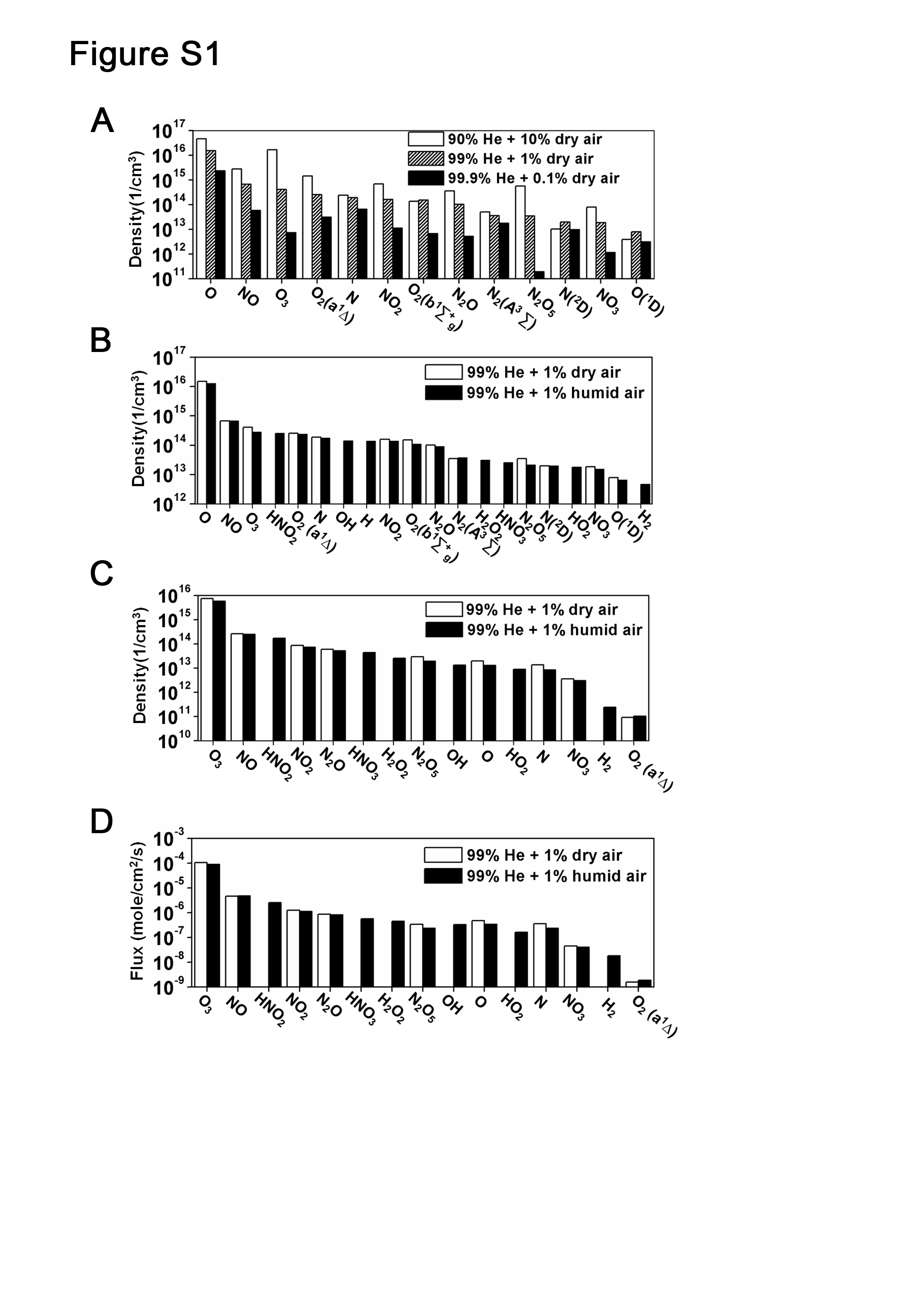

Supplement: Figure S1 — Characterization of NTAPP components and composition. The horizontal axis shows the species names, and the vertical axis shows the density, flux, and mole number. (A) ROS density inside the plasma device without water vapor for the variation of partial fraction of He gas. (B) The comparison of the effect of water vapor with 1% air added in 99% helium. The mole fraction of water vapor is 1%, which corresponds to 30% of relative humidity at room temperature. (C) The amount of ROS diffused into the air after 0.1 ms to reach the surface of dishes containing cells. (D) The mole number of ROS arriving at the dish per unit time and unit area. (TIF) [file pone.0091947.s001.tif]

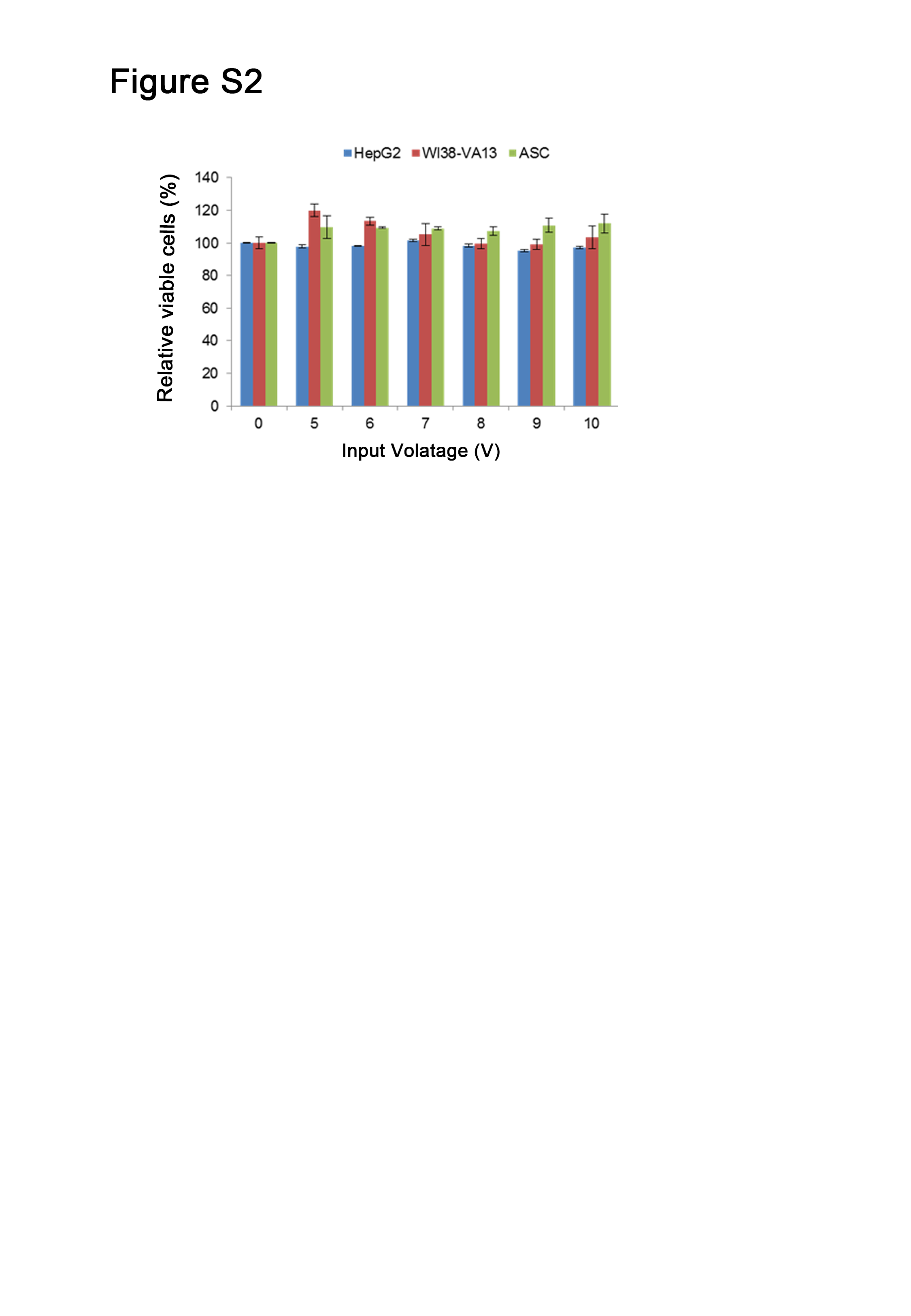

Supplement: Figure S2 — Establishment of optimal exposure conditions for NTAPP. HepG2, WI38-VA13, and adipose tissue-derived stem cells were once exposed for 1 min to NTAPP of various input voltages with 5 SLM (input gas flow ratio to generate NTAPP: standard liter per minute) and 3 cm (the distance between NTAPP to the cell surface) condition. Cells were further incubated for 24 h after NTAPP exposure, and viable cells were quantified with MTT assays. The relative percentages of viable cells were plotted compared with the untreated cells. Data are shown as the mean ± SEM from three independent experiments. (TIF) [file pone.0091947.s002.tif]

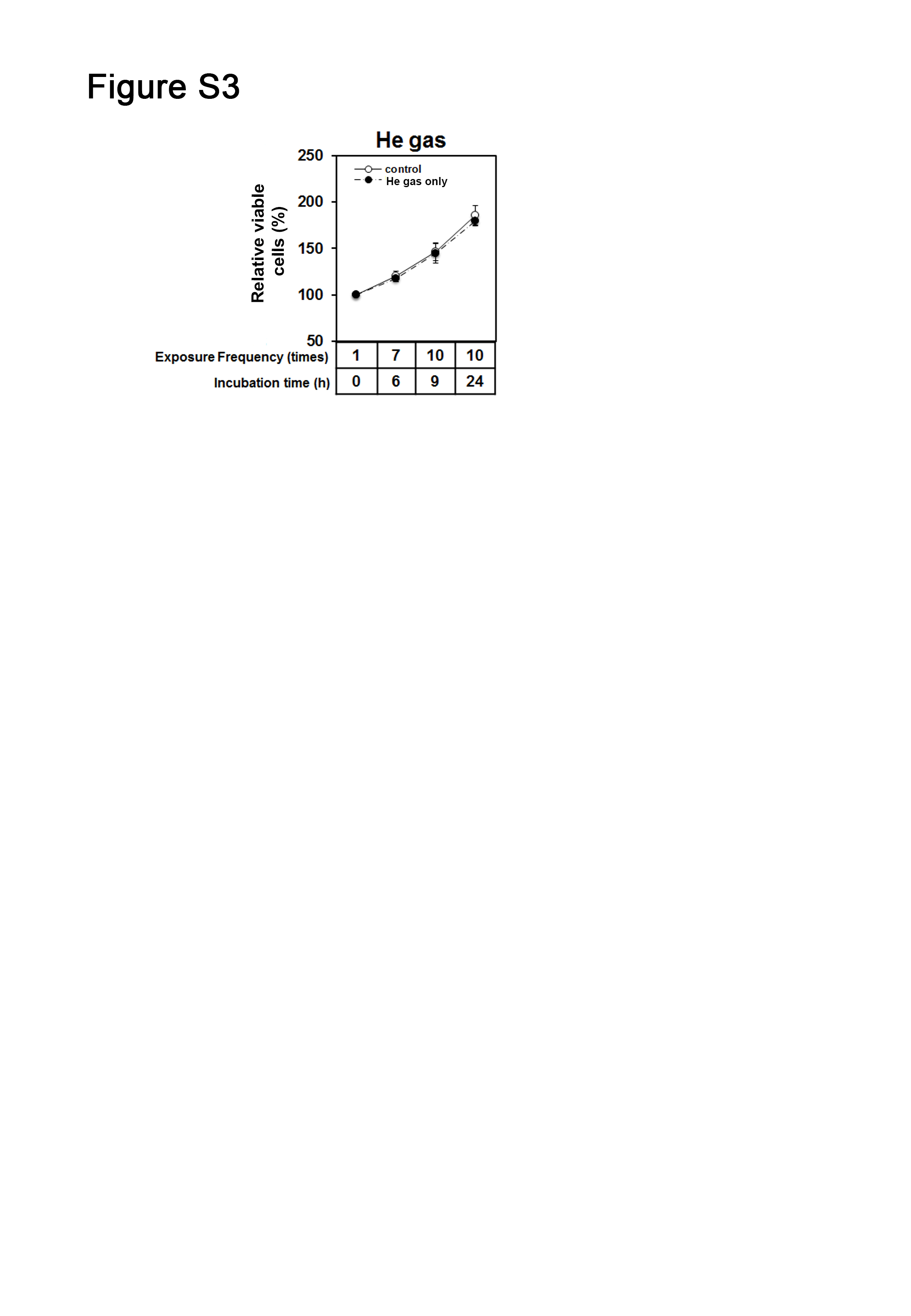

Supplement: Figure S3 — He gas used for NTAPP generation does not affect cell viability. HeLa cells were only exposed to 5 slm He gas for 30 s every h 10 times, and the viable cells were evaluated by MTT assays. The relative percentages of viable cells were plotted compared with the initial cells prior to NTAPP exposure and incubation. Data are shown as the mean ± SEM from three independent experiments. (TIF) [file pone.0091947.s003.tif]

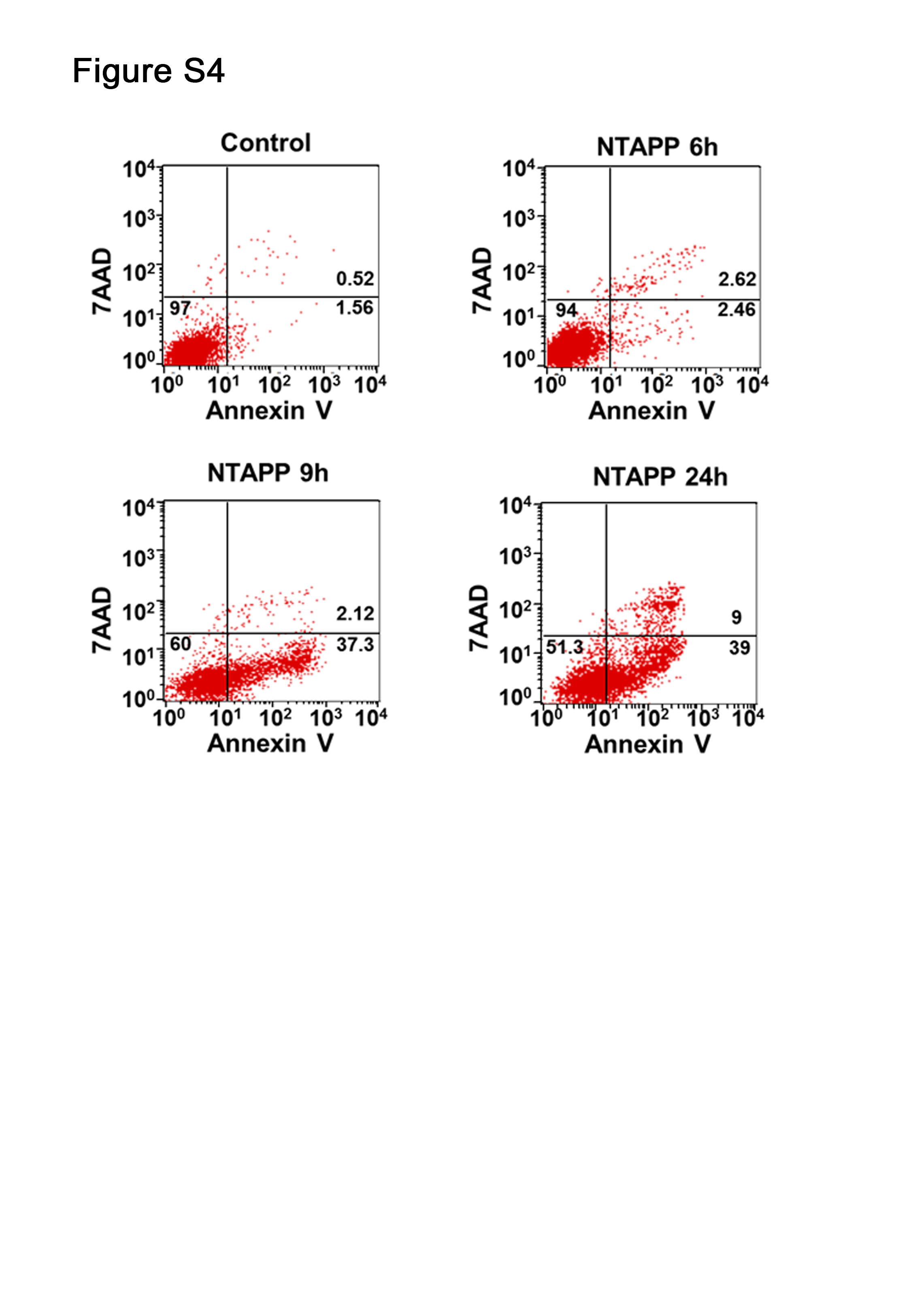

Supplement: Figure S4 — Reduced viability by NTAPP in HeLa cells results from apoptosis. HeLa cells were exposed with 5 V input NTAPP for 30 s every h 10 times, and the induction of apoptosis was determined by flow cytometric analysis with Annexin V-FITC and 7AAD-staining at each indicated exposure frequency. Incubation time indicates the time after the initial NTAPP exposure. The 24 h incubation was prepared with 10 repetitive exposures of NTAPP and further incubation for 15 h. Cells in the lower right quadrant indicate Annexin-positive, early apoptotic cells. The cells in the upper right quadrant indicate Annexin-positive/7AAD-positive, late apoptotic cells. (TIF) [file pone.0091947.s004.tif]

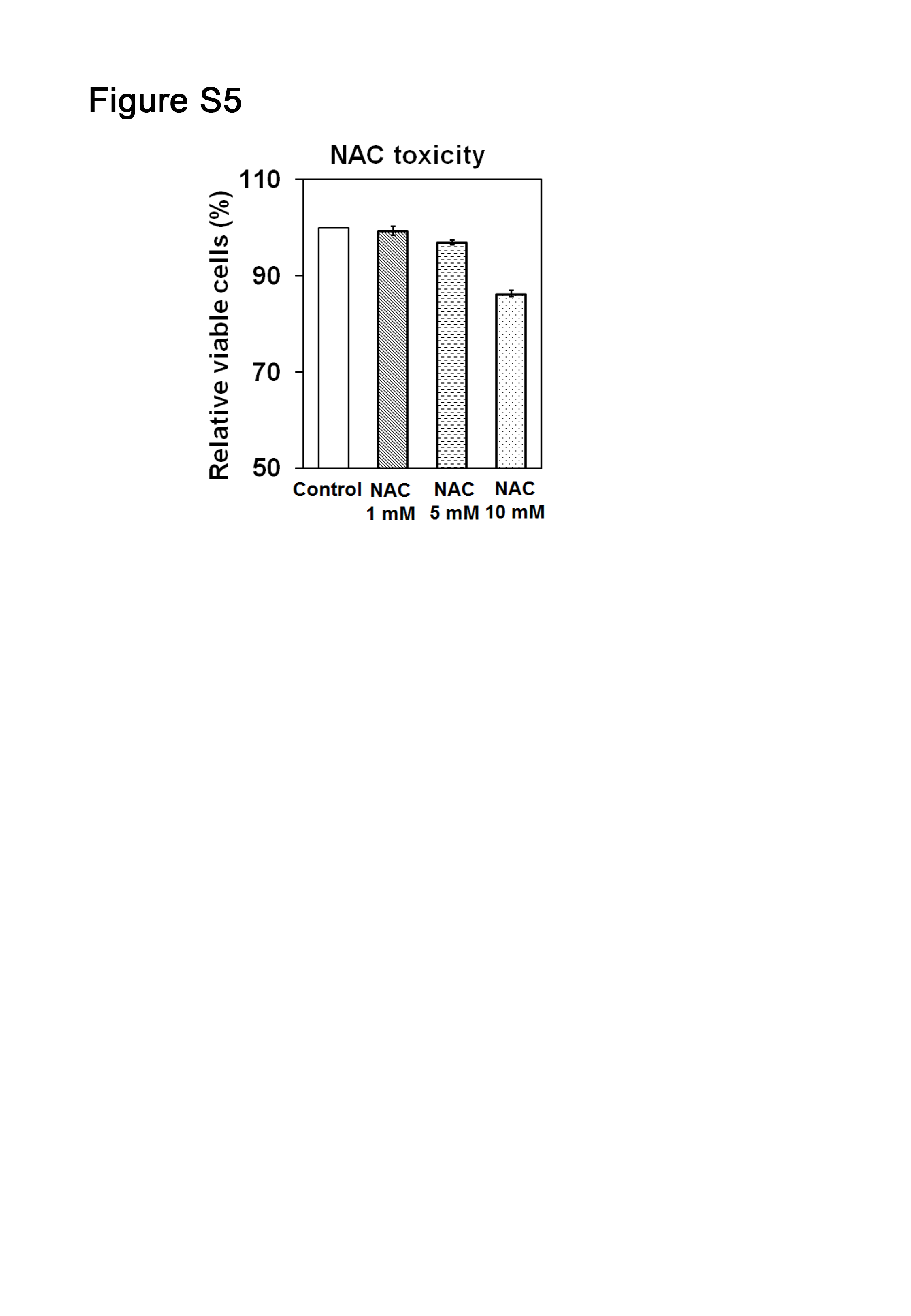

Supplement: Figure S5 — The cytotoxicity of N-acetyl cysteine on HeLa cells. To document the cytotoxicity of the ROS scavenger N-acetyl cysteine (NAC), HeLa cells were incubated in the presence of different concentrations (0, 3, 5, 10 mM) of NAC for 12 h, and viable cells were quantified using MTT assays. The relative percentages of viable cells were plotted compared with the untreated cells. Data are shown as the mean ± SEM from three independent experiments. (TIF) [file pone.0091947.s005.tif]
